# Non-linear dynamics of human locomotion: effects of rhythmic auditory cueing on local dynamic stability

**DOI:** 10.3389/fphys.2013.00230

**Published:** 2013-09-03

**Authors:** Philippe Terrier, Olivier Dériaz

**Affiliations:** ^1^Institute for Research in RehabilitationSion, Switzerland; ^2^Service de Recherche, Clinique Romande de Réadaptation SuvaCareSion, Switzerland

**Keywords:** non-linear gait dynamics, maximal finite-time lyapunov exponents, cued walking, detrended fluctuation analysis, neurorehabilitation, fall risk

## Abstract

It has been observed that times series of gait parameters [stride length (SL), stride time (ST), and stride speed (SS)], exhibit long-term persistence and fractal-like properties. Synchronizing steps with rhythmic auditory stimuli modifies the persistent fluctuation pattern to anti-persistence. Another non-linear method estimates the degree of resilience of gait control to small perturbations, i.e., the local dynamic stability (LDS). The method makes use of the maximal Lyapunov exponent, which estimates how fast a non-linear system embedded in a reconstructed state space (attractor) diverges after an infinitesimal perturbation. We propose to use an instrumented treadmill to simultaneously measure basic gait parameters (time series of SL, ST, and SS from which the statistical persistence among consecutive strides can be assessed), and the trajectory of the center of pressure (from which the LDS can be estimated). In 20 healthy participants, the response to rhythmic auditory cueing (RAC) of LDS and of statistical persistence [assessed with detrended fluctuation analysis (DFA)] was compared. By analyzing the divergence curves, we observed that long-term LDS (computed as the reverse of the average logarithmic rate of divergence between the 4th and the 10th strides downstream from nearest neighbors in the reconstructed attractor) was strongly enhanced (relative change +73%). That is likely the indication of a more dampened dynamics. The change in short-term LDS (divergence over one step) was smaller (+3%). DFA results (scaling exponents) confirmed an anti-persistent pattern in ST, SL, and SS. Long-term LDS (but not short-term LDS) and scaling exponents exhibited a significant correlation between them (*r* = 0.7). Both phenomena probably result from the more conscious/voluntary gait control that is required by RAC. We suggest that LDS and statistical persistence should be used to evaluate the efficiency of cueing therapy in patients with neurological gait disorders.

## Introduction

During walking, individuals are able to voluntarily adjust their gait to external cues, such as floor markers, metronomes, or the moving belt of a motorized treadmill. External spatial or temporal stimuli could facilitate movement: namely, cued walking exhibits positive effects on various gait characteristics of neurologically impaired patients (Thaut and Abiru, 2010), such as patients with Parkinson's Disease (PD; Nieuwboer et al., 2007), or stroke (Thaut et al., 2007; Roerdink et al., 2009). In PD patients, synchronizing steps with an external rhythmic stimulus (Rhythmic Auditory Cueing, RAC), significantly improves walking speed, stride length (SL), and cadence (Lim et al., 2005). Similarly, it has been suggested that a treadmill could act as an external cue to enhance gait rhythmicity and reduce gait variability (speed cueing; Frenkel-Toledo et al., 2005), which may improve SL, maximal speed and balance (Bello et al., 2013). Combining several cues together seems to provide further enhancements: treadmill training associated with auditory and visual cues might give better results than more conventional treatments (Frazzitta et al., 2009).

Although empirical evidence supports the use of cued walking in neurorehabilitation practice, many aspects of the underlying neurophysiological mechanisms are not yet fully understood (Bello and Fernandez-Del-Olmo, 2012). It is thought that a continuous control of kinematic fluctuations is required in order to minimize energy expenditure and fall risks (Zarrugh et al., 1974; Bauby and Kuo, 2000; Donelan et al., 2001). Those continuous optimizations likely imply both feedforward (from internal models) and feedback (from sensory inputs) mechanisms (Kuo, 2002), which require low attentional demands and are highly automated: the existence of specific structures at the spinal level (central pattern generators) is strongly suspected (Dimitrijevic et al., 2006). In contrast, synchronizing movement with rhythmic auditory cues requires complex supraspinal mechanisms, which induce increased neuronal activity in sensorimotor cortex, supplementary motor area, premotor cortex, inferior parietal cortex, basal ganglia, and cerebellum (Repp and Su, 2013).

While the neurophysiological mechanisms of cued walking have not yet been fully characterized, it is evident that synchronizing gait with external stimuli mobilizes specific supraspinal/cortical processes, which add to basic gait control. That activation modifies the stride-to-stride fluctuation pattern of basic gait parameters. Indeed, an interesting feature of gait control is that reasonable deviations from the mean persist across subsequent strides: time series of stride time (ST), SL, and stride speed (SS) exhibit substantial long-range autocorrelation (statistical persistence), probably due to feedback loops in gait control (Hausdorff et al., 1995; Terrier et al., 2005). In other words, a larger stride as compared to average SL is more likely to be followed by subsequent larger strides. In overground walking, RAC induces a strong anti-persistence in ST time series (Terrier et al., 2005; Sejdic et al., 2012), while a persistent pattern is conserved in SL and SS (Terrier et al., 2005). Anti-persistence means that deviations in one direction are statistically more likely to be followed by subsequent deviations in the opposite direction (i.e., longer strides are more likely to be followed by shorter strides). Similarly, treadmill walking (speed cueing) seems to induce an anti-persistent pattern in SS only (Dingwell et al., 2010), while ST and SL remain persistent. When both treadmill and RAC are combined, all three parameters (ST, SL, and SS) are anti-persistent (Terrier and Dériaz, 2012). The anti-persistent pattern could be induced by fast over-correction of deviations in the controlled variable, which results in continuous oscillations around target values (Dingwell et al., 2010; Terrier and Dériaz, 2012).

The local dynamic stability (LDS) is another approach that has been proposed to characterize non-linear properties of gait variability. It evaluates the faculty to maintain steady progression despite the constant presence of small internal control errors or small external disturbances. Gait LDS can be characterized using the maximal Lyapunov exponent, which is a parameter that assesses how fast a system diverges from neighboring points in a state space that characterizes the dynamic of the system (Brown, 1996; Dingwell and Cusumano, 2000; Dingwell, 2006; Terrier and Dériaz, 2011). Modeling studies (Roos and Dingwell, 2010; Bruijn et al., 2011), as well as experimental studies in healthy individuals (McAndrew et al., 2011; Van Schooten et al., 2011; Hak et al., 2012), provide some evidences that LDS is actually related to fall risk. Furthermore, recent clinical studies observe that older people at risk for falling exhibited lower LDS (Lockhart and Liu, 2008; Toebes et al., 2012). A recent review concludes that LDS is among the best gait stability indexes for fall risk prediction (Bruijn et al., 2013).

**Figure A2:**
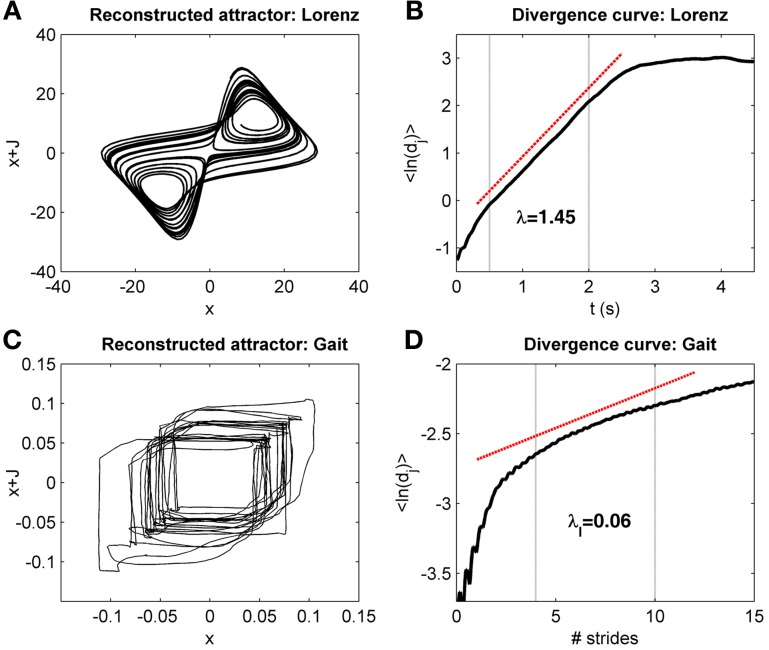
**Divergence curves**. **(A)** Shows the reconstructed Lorenz attractor build with the same parameters as in Rosenstein et al. (1993). The corresponding divergence curve (i.e., the logarithmic average divergence as a function of time, Equation A6) is presented in the **(B)**. The “prominent” linear region between 0.5 and 2 s is highlighted by the vertical dotted lines. The linear fit to the curve in that region is highlighted by the red dotted line, and the slope corresponds to the maximal Lyapunov exponent (Equations A5 and A6), λ = 1.45. The true Lyapunov exponent for the Lorenz attractor is 1.5. The reconstructed attractor for gait data (mediolateral signal, Figures 1, A1) is displayed in **(C)**. **(D)** Shows the corresponding divergence curve, with time normalized by the average stride duration. The time scale for the computation of the long-term LDS (4–10 strides) is shown with the vertical dotted lines. The linear fit is displayed (red dotted line), with the slope value (divergence exponent λ_L_).

While it is still uncertain whether LDS could constitute a relevant and usable proxy for fall risk, LDS is a valid non-linear measure of gait variability based on sound theoretical background (Dingwell, 2006; Stergiou and Decker, 2011). It can therefore serve to highlight potential changes in the fluctuations of continuously measured kinematics variables induced by various conditions, such as cued walking. The method implies to measure the average divergence rate among neighboring trajectories (see appendix A). Computing the short-term divergence (short-term LDS) corresponding to one stride or one step is likely the most relevant time scale to estimates gait stability (Bruijn et al., 2013). However, a longer time scale (long-term LDS, classically between the 4th and the 10th stride after the initial perturbation, see Figure A2) has been also proposed (Dingwell and Cusumano, 2000). Studies have highlighted an opposite responsiveness between short-term and long-term LDS in experiments that artificially destabilized healthy individuals (McAndrew et al., 2011; Van Schooten et al., 2011). Moreover, it has been shown that treadmill walking induces higher short-term and long-term LDS as compared with overground walking (Dingwell et al., 2001; Terrier and Dériaz, 2011). On the other hand, it has been recently observed that RAC induced a higher long-term LDS in overground walking, with no significant change in short-term LDS (Sejdic et al., 2012). The significance of those findings remains unclear and deserves further investigations. In particular, the combination of RAC and treadmill walking and its effect on LDS has not been studied.

The statistical persistence/anti-persistence and LDS of walking characterize distinct aspects of gait control (Terrier and Dériaz, 2011). Assuming an autoregressive stochastic process, statistical persistence quantifies temporal dynamics of discrete events (ST, SL, SS) over hundreds of consecutive strides, and serves to characterize the feedbacks in locomotor control (Dingwell et al., 2010; Terrier and Dériaz, 2011, 2012). Assuming a chaotic system, LDS quantifies temporal dynamics in continuous signals (acceleration, speed, position) and assesses the degree of resilience of motor control to small perturbations over shorter timescales (Dingwell, 2006; Roos and Dingwell, 2010). Although these two indexes seem loosely related from a theoretical point of view, they are both responsive to cued walking (Dingwell et al., 2001; Terrier and Dériaz, 2011; Sejdic et al., 2012). A treadmill experiment revealed a strong correlation between long-term LDS and statistical persistence (Jordan et al., 2009), but this was contradicted by others (Terrier and Dériaz, 2011). Therefore, more results are needed to assess whether correlations exist between LDS and statistical persistence and to clarify the mechanism behind such a potential association.

LDS has been computed from several kinematics parameters (e.g., accelerations, joint angle) using various measurement technique (e.g., video analysis, accelerometer, goniometer). Each method has its own drawbacks and advantages. In order to analyze persistent pattern in time series of ST and SL, we proposed the use of a treadmill, instrumented with foot-pressure sensors aimed at dynamic plantar pressure assessment (Terrier and Dériaz, 2012). Because it allows the continuous measure of the position of the center of pressure, it would be possible to assess the LDS in parallel. The main advantage is that LDS and scaling exponents (SL, ST, and SS) would be directly measured with the same instrument.

The objective of the present study was to analyze the effect of RAC on LDS in healthy, middle-aged individuals walking on a treadmill. To estimate LDS, an innovative technique based on the measure of the center of pressure was tested. Both short-term and long-term LDS were assessed: the aim was to compare the responsiveness to RAC at different time scales. The hypothesis was that the increased control over the gait while combining both treadmill and RAC would result in a more locally stable gait. In addition, the correlation between LDS and statistical persistence [data from a companion article (Terrier and Dériaz, 2012)] was evaluated. The overall aim is to determine whether LDS could constitute a relevant index for studying cued walking.

## Methods

The present study is based on raw data obtained in a previous study (Terrier and Dériaz, 2012). Please refer to this article for further information about the experimental procedure.

### Participants

Twenty healthy subjects (10 females, 10 males) took part in the study. The participants' characteristics were mean (SD): age 36 years (11), body mass 71 kg (15), and height 171 cm (9). The experimental procedure was approved by the local ethics committee (Commission Cantonale Valaisanne d'Ethique Médicale, Sion, Switzerland).

### Experimental procedure

The testing sessions consisted of two series of three 5 min 30 s treadmill walking: 30 s of habituation to the speed, and 5 min of measurement. Treadmill speeds imposed on the subjects were: Preferred Walking Speed (PWS), 0.7 × PWS (low speed) and 1.3 × PWS (high speed). The speed sequence was randomly attributed. The trials with the “metronome” condition (treadmill + RAC) were performed at the same speeds as the first 3 trials. The imposed cadences were the preferred cadences, which were measured during the first trials without metronome.

The measurement device was a motorized treadmill (FDM-TDL, Scheinworks/Zebris, Schein, Germany), instrumented with foot-pressure sensors (100 Hz sampling rate, 128 × 56 pressure sensors on a 108.4 × 47.4 cm grid). A “movie” of the feet pressure on the treadmill belt was obtained (as illustration, see online supplementary materials, video S1). The raw data consisted for each trial of 30,000 frames of 7,168 points. They were exported for subsequent analysis with Matlab (Mathworks, MA, USA). Complementary statistical analysis was realized with Stata (StataCorp, TX, USA).

### Data analysis

**Figure 1:**
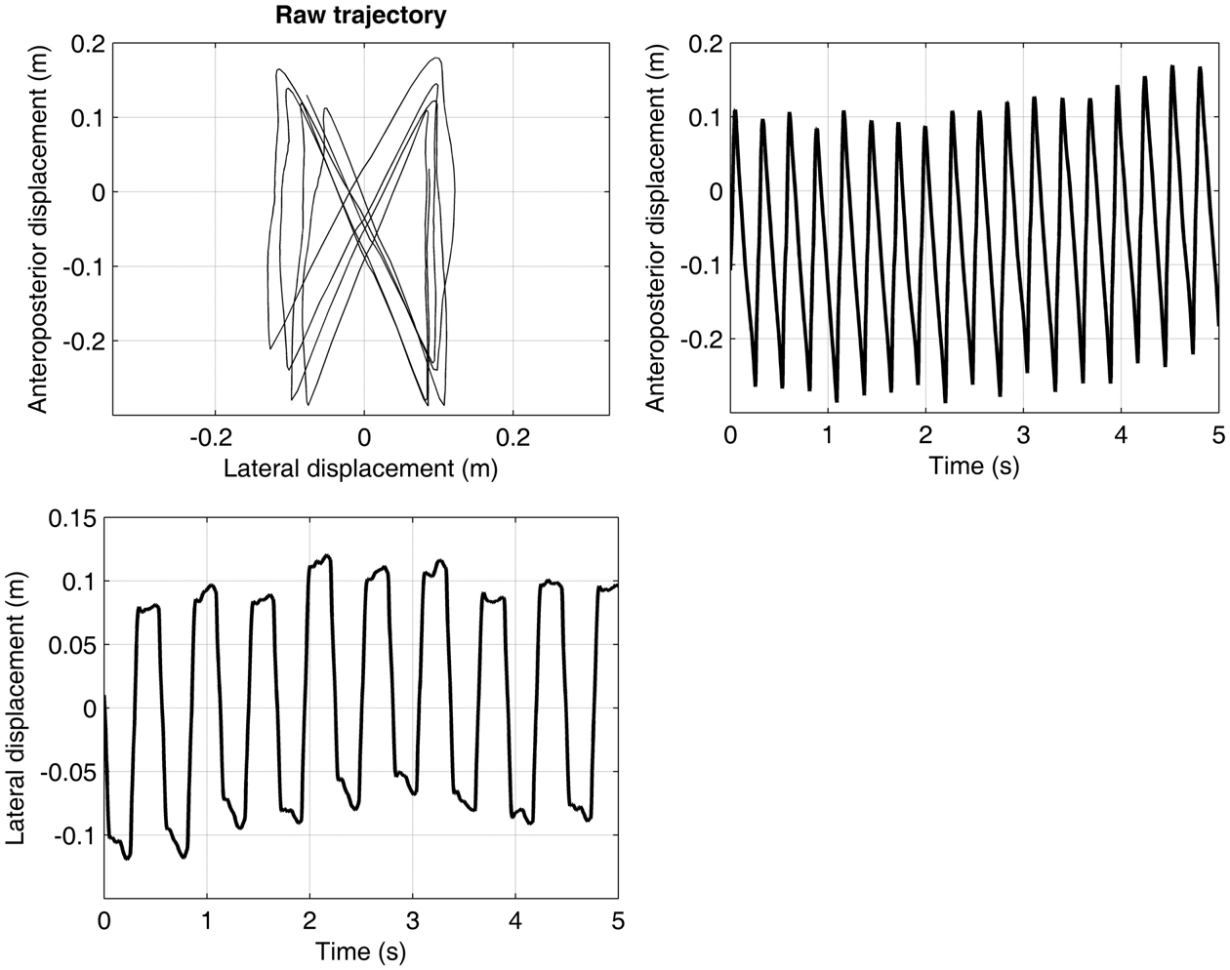
**Trajectory of the center of pressure**. A participant walked at preferred walking speed (1.25 m·s^−1^) during 5 min. on an instrumented treadmill, which measured the feet pressure on the treadmill belt. The trajectory of the center of pressure was computed (barycenter method). Only 5 s are shown. The upper-left panel shows the raw trajectory. The upper-right and lower panels show the decomposition of the raw trajectory in, respectively, the anteroposterior and the mediolateral directions.

The continuous trajectory of the center of pressure was computed as the weighted average of the pressure data, and using the standard method for determining the barycenter (Sum of mass × position)/(Sum of mass). The two axes of the trajectory consisted of an anteroposterior (AP) component (along the direction of the displacement of the treadmill belt) and a mediolateral component (ML, perpendicular to the displacement). Figure 1 presents a typical plot of center of pressure trajectory, with the corresponding AP and ML signals. The raw trajectory can be also seen in the video provided in the online supplementary material.

The raw 100 Hz signals were filtered down to 50 Hz in order accelerate the subsequent steps of data analysis; an eighth-order low pass Chebyshev Type I filter was used, which filtered the signal in both the forward and reverse directions to remove all phase distortion (Matlab command *decimate*). Step Frequency (SF), and thus average step duration, was assessed by calculating the Fast Fourier Transform of the AP signal. Then, a duration corresponding to 175 strides was selected from the raw signals. The resulting segments, whose length depended upon the SF of each participant at each speed condition, were time-standardized to a uniform length of 10,000 samples, by using a polyphase filter implementation (Malab command *resample*).

**Figure 2:**
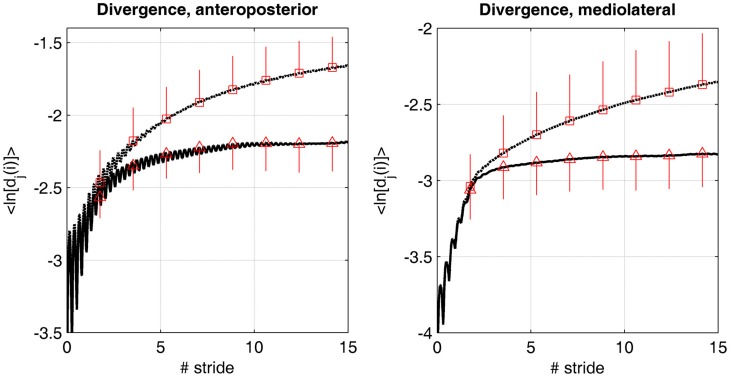
**Divergence curves**. The average logarithmic divergence (<ln[d_*j*_(i)]>) in anteroposterior and mediolateral directions was measured in the reconstructed state space of the center of pressure trajectory (50 Hz sampling rate), in 20 individuals walking at preferred walking speed. 175 consecutive strides were analyzed, normalized at 10,000 samples. The value at each time (50 Hz) was averaged across the subjects (*N* = 20). Time was normalized by the average stride time (1.14 s). Discontinuous lines (squares) are the results for the treadmill only condition. Continuous lines (triangles) are the results for the dual cueing condition (treadmill + rhythmic auditory cueing). Mean value at 100, 200, 300, 400, 500, 600, 700, and 800 samples are shown (squares and triangles) with the corresponding SD (vertical lines, *N* = 20).

The method for quantifying LDS has been described in many articles (Dingwell and Cusumano, 2000; Lockhart and Liu, 2008; Terrier and Dériaz, 2011). More theoretical information is provided in the appendix A at the end of the article. The state space was reconstructed according to the Takens' theorem, as classically applied in gait dynamics studies (Dingwell and Cusumano, 2000). The time delay and the embedding dimension were assessed by the average mutual information (AMI) function and global false nearest neighbors (GFNN) analysis, respectively. A time delay of 15 and 18 samples, respectively, was used for the ML and AP directions. A constant dimension of six was set for all the directions. These values corresponded to the average results of the AMI and GFNN analyses. In order to illustrate the influence of RAC on the divergence dynamics, the mean logarithmic divergence curve (see Figure A2) was computed by averaging each sample across participants (*N* = 20). In addition, Standard Deviation (SD) was computed at seven discrete points (Figure 2). As in other studies (Dingwell and Cusumano, 2000; Dingwell et al., 2001; Yakhdani et al., 2010; Van Schooten et al., 2011), two divergence exponents were computed: short-term LDS over the timescale corresponding to the first step (λ_S_) and long-term LDS (λ_L_) over the timescale between the 4th and 10th strides.

### Statistics

**Figure 3:**
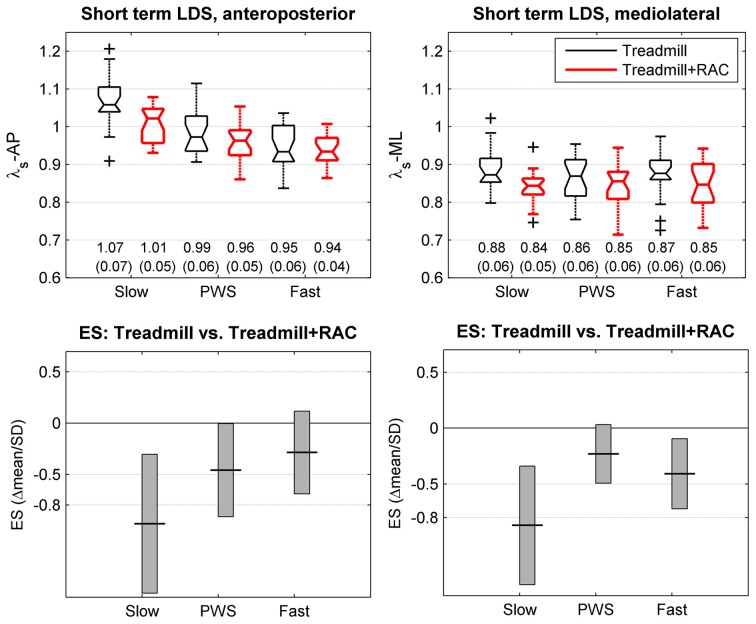
**Short-term local dynamic stability (LDS)**. Twenty healthy subjects walked 3 × 5 min on an instrumented treadmill without (thin lines, black) and with Rhythmic Auditory Cueing [RAC, (metronome), thick lines, red] at their preferred cadence for the given speed. The center of pressure trajectory over 175 consecutive strides was analyzed along the anteroposterior and mediolateral axes. Short-term LDS is computed from the rate of logarithmic divergence over one step (finite time Lyapunov exponents, λ_S_). Selected speeds were Preferred Walking Speed (middle, PWS), 0.7 × PWS (left, Slow) and 1.3 × PWS (right, Fast). The range of individual results (*N* = 20) is presented with notched boxplots. + signs represent outliers. Printed values are mean (SD). Bottom panels show the effect size (ES) of the auditory cueing [i.e., the mean difference normalized by SD (Hedges's g)]. Vertical boxes are the 95% confidence intervals for the effect size estimations.

**Figure 4:**
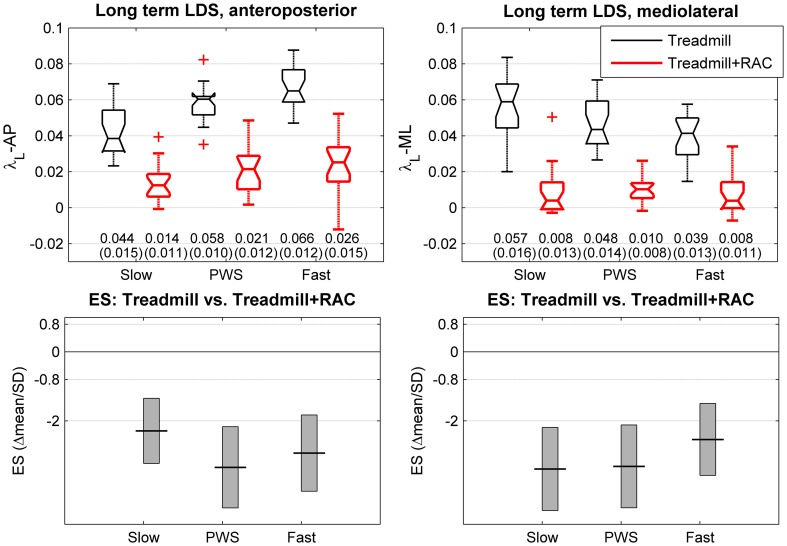
**Long-term local dynamic stability (LDS)**. Twenty healthy subjects walked 3 × 5 min on an instrumented treadmill without (thin lines) and with Rhythmic Auditory Cueing [RAC, (metronome), thick lines at their preferred cadence for the given speed]. The center of pressure trajectory over 175 consecutive strides was analyzed along the anteroposterior and mediolateral axes. Long-term LDS is computed from the rate of logarithmic divergence among the 4 to 10th consecutive strides (finite time Lyapunov exponents, λ_L_). Selected speeds were Preferred Walking Speed (middle, PWS), 0.7 × PWS (left, Slow) and 1.3 × PWS (right, Fast). The range of individual results (*N* = 20) is presented with notched boxplots. + signs represent outliers. Printed values are mean (SD). Bottom panels show the effect size (ES) of the auditory cueing [i.e., the mean difference normalized by SD (Hedges's g)]. Vertical boxes are the 95% confidence intervals for the effect size estimations.

We analyzed four dependent variables: (1) short term LDS in the anteroposterior direction (λ_S_-AP); (2) short term LDS in the mediolateral direction (λ_S_-ML); (3) long term LDS in the anteroposterior direction (λ_L_-AP); (4) long term LDS in the mediolateral direction (λ_L_-ML). The independent variables were speed (3 level) and cueing condition (treadmill and treadmill + RAC), but in the present study we were interested mainly in the effect of RAC. The descriptive statistics of dependent variables consisted of the mean and standard deviation, separately for each independent variable (Figures 3, 4). In addition, the spread of the individual results were presented by using notched boxplots (median and quartiles, *N* = 20 participants, Figures 3, 4). Standardized Effect Size (ES = delta(mean)/SD_*pooled*_, i.e., Hedges's g) was computed in order to describe the strength of the effect of RAC (Cohen, 1992; Nakagawa and Cuthill, 2007). The precision on the effect sizes was estimated with 95% Confidence Intervals (CI). CI were ±1.96 times the asymptotic estimates of the standard error of g. Graphical representations of ES and corresponding CI are shown for each variable and speed condition (Figures 3, 4). Arbitrary thresholds for medium (0.5), large (0.8), and huge (2) effects (Cohen, 1992) were used in order to ease the interpretation. It should be reminded that the analysis of ES and CI is strictly equivalent to the paired *t*-test. Furthermore, in order to minimize type I error risk induced by the multiple comparisons (3 different speeds, 2 directions), the analysis was completed using a multivariate comparison test (Hotelling's *T*-squared test) separately for long-term and short-term LDS, which is similar to omnibus ANOVA testing: The null hypothesis H0 was that the mean differences (treadmill + RAC minus treadmill) were equal to zero.

**Figure 5:**
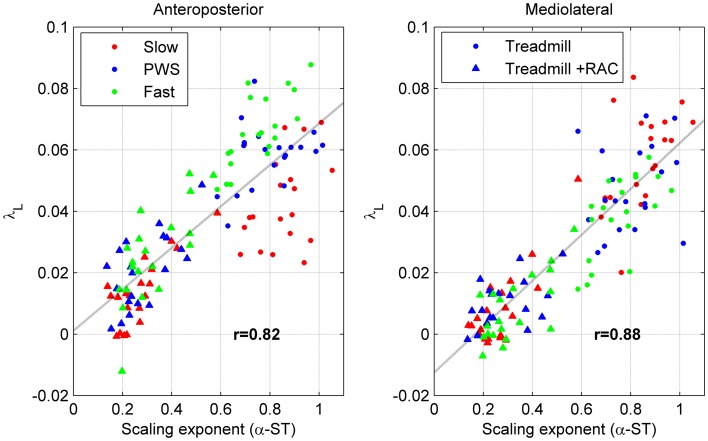
**Scatter plot of statistical persistence and long-term local dynamic stability (LDS)**. Twenty subjects walked on a treadmill at slow speed (red), preferred walking speed (PWS, blue), and fast speed (green) without (circles) and with (triangles) rhythmic auditory cueing (*N* = 120). Detrended fluctuation analysis was used to determine the scaling exponents of the time series of stride time (α-ST, see Appendix B), which are plotted along the horizontal axis. Divergence exponents (λ_L_, see Figure 4) are plotted along the vertical axis. The discontinuous line is the best linear fit (least squares method). Values are the Pearson's correlation coefficients (r).

Next, we are interested in comparing the LDS results with persistence results, which were presented in the above-mentioned companion article (Terrier and Dériaz, 2012): detrended fluctuation analysis (DFA) was used to characterize statistical persistence using scaling exponents (α). The results are summarized in the appendix B. A scatterplot was used to illustrate the potential association between scaling exponents (α) and divergence exponents (λ). In particular, we plotted (Figure 5) the overall DFA results for ST (α-ST in 20 subjects × 3 speeds × 2 conditions = 120 points) vs. the overall long-term LDS results (λ_L_). The Pearsons's *r* correlation coefficient was computed, not only for the λ_L_ vs. α-ST association, but also for λ_L_ vs. α-SL, λ_S_ vs. α-ST, and λ_S_ vs. α-SL.

We completed the analysis using Canonical Correlations Analysis (CCA), after removing potential speed effects. The main advantage of CCA is that it reduces the risk of type I errors that increase when multiple correlations are performed (Hair et al., 2010). First, a linear regression was computed between the average speed and each dependent variable, separately for each cueing condition (*N* = 60). The spread of the average speeds among individuals can be found in another companion article (Terrier, 2012). Then, the residuals of the linear regression were computed by subtracting the predicted values from the data (observed response minus predicted response). The residuals reflect the remaining variance when linear speed effects are removed. In other words, the LDS and DFA results were controlled for the speed covariate. Consequently, the risk that speed would bias the results was minimized, and the sample size (*N* = 60) was maximized. The CCA is a multivariate statistical method that assesses the strength of association between two sets of variables (Hair et al., 2010). The relationship (canonical function) between two linear composites (variates) is computed. The canonical correlation coefficient expresses the strength of the relationship between the two variates that compose the canonical function. Three sets of variables were defined for each condition: from the results of the present study, set#1: [λ_S_-AP; λ_S_-ML], set#2 [λ_L_-AP; λ_L_-ML]; from the results of the previous study, set #3 [α-ST; α-SL; α-SS]. Two CCAs were realized for each condition, set#1 vs. set#3 and set#2 vs. set#3. Given the size of the sets, two orthogonal canonical correlation coefficients were obtained. The significance of those canonical correlations (i.e., *r* <> 0) was assessed with the Wilks' lambda statistics. Furthermore, the analysis was completed with redundancy results, which express the amount of variance in one set explained by the linear composite (canonical variate) of the other set.

## Results

Figure 2 shows the average logarithmic divergence <ln [*d*_*j*_(*i*)]> in both conditions (i.e., treadmill and treadmill + RAC). A very different divergence regime is observed. While very few differences are evident over the first stride (short-term LDS), the curve reaches a plateau faster under the treadmill + RAC condition.

The descriptive statistics for the short term LDS (λ_*s*_) are shown in the upper panels of Figure 3. The results of the multivariate *T*^2^ test revealed that a significant effect of RAC is likely (*T*^2^ = 40, *p* = 0.007), but the average relative change is small (−3%). A lower λ (lower divergence rate) signifies that the LDS was higher. The partial ES results (Figure 3, lower panels) are contrasted, but it seems that a relevant effect (increased LDS) is effective at lower speeds (λ_S_-AP: −6%, ES: −1.1; λ_S_-ML: −4%, ES: −0.84). At PWS, the results are λ_S_-AP: −4%, ES: −0.44; λ_S_-ML: −1%, ES: −0.24. At fast speeds, the results are λ_S_-AP: −1%, ES: −0.26; λ_S_-ML: −3%, ES: −0.44.

The descriptive statistics for the long term LDS (λ_L_) are shown in the upper panels of Figure 4. The results of the multivariate *T*^2^ test shows a highly significant effect of RAC (*T*^2^ = 415, *p* < 0.0001), with a large increase in LDS (73% in average). The partial ES results (Figure 4, lower panels) revealed very large effects (lower λ ≥ higher LDS) across all directions and speeds: slow speed: λ_L_-AP: −68%, ES: −2.3; λ_L_-ML: −86%, ES: −3.4; PWS: λ_L_-AP: −64%, ES: −3.3; λ_L_-ML: −80%, ES: −3.3; fast speed: λ_L_-AP: −61%, ES: −2.9; λ_L_-ML: −80%, ES: −2.5.

The Figure 5 globally compares the long-term LDS results with the DFA results (λ_L_ vs. α-ST, *N* = 120). Because both variables are responsive to RAC (compare Figure 4 and Table B1), the treadmill + RAC points (triangles) are logically collated in the lower-left quadrants, and the treadmill-only points (circles) are found in the upper-right quadrants. As a result, a high correlation was found (0.82 and 0.88). High correlation coefficients are also fund when λ_L_ and α-SL are compared (*r* = 0.81 and 0.87). In contrast, short-term LDS is poorly correlated with α-ST (*r* = 0.21 and 0.23) and with α-SL (*r* = 0.16 and 0.16), because the response of short-term LDS to RAC is lower.

Table 1 summarizes the results of the CCA, which analyze the association between scaling exponents and divergence exponents separately for both conditions (treadmill and treadmill + RAC, *N* = 60). The two canonical orthogonal functions resulted in two canonical correlation coefficients, which are presented in the first and second columns, with corresponding *p*-values in the third and fourth columns. Only the redundancy results of the first canonical function are shown in the last two columns. Regarding results for the short-term LDS (set#1 vs. set#3), the hypothesis that a correlation exists with statistical persistence should be rejected. Indeed, low (0.09–0.30), not significant, correlation coefficients are observed and the redundancy results show that the canonical functions explain a very small part of the variance in the other set (3–6%). On the contrary, a relevant association between the long-term LDS and the statistical persistence is likely, especially under the treadmill + RAC condition (*p* < 0.001). In addition, the redundancy results of the first canonical function reveal that a substantial part of the variance in one canonical variate is explained by the other canonical variate (20–53%).

**Table 1:** **Canonical correlation analysis (CCA)**.

		**Canonical correlation coefficients**		***p*-values (Wilks') (lambda statistics)**		**Redundancy first (canonical function)**	
Treadmill only	Short-term LDS vs. Scaling exponents	0.30	0.09	0.32	0.42	3%	5%
	Long-term LDS vs. Scaling exponents	**0.57**	0.33	0.00	0.24	20%	20%
Treadmill + RAC	Short-term LDS vs. Scaling exponents	0.29	0.19	0.20	0.34	3%	6%
	Long-term LDS vs. Scaling exponents	**0.83**	0.26	0.00	0.14	41%	53%

Scaling exponents (α) evaluate the statistical persistence present in the time series of stride time (ST), stride length (SL) and stride speed (SS). The local dynamic stability assesses the local divergence (λ) of the trajectory of the center of pressure in anteroposterior (AP) and mediolateral (ML) directions. Two CCAs were performed for each cueing condition: (1) between short-term LDS (two variables, λ_s_-AP and λ_s_-ML) and scaling exponents (3 variables, α-ST, α-SL, and α-SS); (2) between long-term LDS (two variables, λ_L_-AP and λ_L_-ML) and scaling exponents (3 variables, α-ST, α-SL, and α-SS). All the seven variables were normalized with respect to walking speed prior to CCA (N = 60, i.e., 3 imposed speed times 20 participants for each variable). Significant values (*p* <*0.05*) are highlighted in bold. See text for more precision on the CCA method.

## Discussion

By measuring the trajectory of the center of pressure on a motorized treadmill, the objective of the present study was to analyze the responsiveness of LDS to RAC in healthy individuals. RAC slightly increased short-term LDS, with an effect that was especially evident at slow speeds. On the other hand, a huge effect of RAC on long-term LDS was observed: LDS was largely increased for all speeds and directions. Correlation results revealed that a relevant association (positive correlation) between long-term LDS and statistical persistence (scaling exponent α) is likely, especially under the “treadmill + RAC” condition. On the contrary, an association between statistical persistence and short-term LDS is very unlikely.

### Methodological considerations

As far as we know, the present study proposes for the first time computing LDS from the trajectory of the center of pressure obtained from an instrumented treadmill. As illustrated in Figure 1 and in the online movie (supplementary material), small deviations in the trajectory is evident from one stride to the next, which are the manifestation of the continuous adjustments that the motor control performs to maintain stable gait. Other authors have used the center of pressure in gait stability studies (Day et al., 2012), for instance to analyze how motor control reacts to large external perturbations (Hof et al., 2010). The center of pressure trajectory seems therefore a relevant parameter, from which LDS can be computed. Moreover, the results of the present study are comparable to those of a recent study that analyzed the response of LDS to RAC in overground walking (Sejdic et al., 2012), which supports the fact that the method correctly assesses the LDS.

As in other recent LDS studies (Yakhdani et al., 2010; Van Schooten et al., 2011; McAndrew Young and Dingwell, 2012), this study used a normalized sample size (10,000) and a normalized number of strides (175). It also employed uniform time delays and dimensions. As proposed by others (Yakhdani et al., 2010; Van Schooten et al., 2011), this study computed short-term LDS over one step, and not one stride. Regarding short-term LDS, the total variance (Figures 3, 4), which is the combination of the actual biological inter-individual variability, the actual intra-individual variability and the measurement error, was rather low: expressed as CV (SD/mean), it lies between 5 and 8%. In comparison, in a previous treadmill study that used trunk accelerometry and a less standardized methodology (Terrier and Dériaz, 2011), we observed an average CV of 21% for short-term LDS. Because both studies included the same number of subjects who were sampled from the same population, the difference is very likely the measurement error. Consequently, the combination of standardized procedures and use of the center of pressure trajectory probably makes it possible to obtain a lower measurement error and thus higher reliability, which increases the statistical power and reduces the risk of type II errors.

### Effects of rhythmic auditory cueing

The logarithmic divergence curves, such as presented in Figure 2, were strikingly modified by RAC. It is known that with the Rosenstein's algorithm a plateau is reached when the divergence cannot further grow because of the limits of the attractor (Figure A2). In other words, the trajectories in the state space form a flow, which is bounded. As hypothesized in the introduction, RAC enabled specific sensory-motor synchronizing processes: this additional control probably narrowed the maximal bounds in the state space. Because reconstructed phase space reflects the dynamical comportment of gait, a narrower flow in the attractor indicates that RAC restricts the dynamical range, which is employed by motor control during walking. The modification of the attractor bounds is logically also reflected in long-term LDS results, because it is computed from the slope close to the plateau (Figures 2, A2). Thus, long-term LDS was strongly enhanced (lower λ_*l*_, ES > 2, relative change 73%). On the other hand the change in short-term LDS was smaller (ES −0.55, 3% relative change). The study by (Sejdic et al., 2012) highlighted the same findings in overground walking, which indicate that the LDS change is not solely due to the interaction between treadmill and RAC.

### Comparative responsiveness of short-term and long-term local dynamic stability

The results suggest that a specific modality of gait control (i.e., the synchronization with an external cue) may affect differentially short-term and long-term LDS through a modification of divergence curves.

Three theoretical studies based on artificial gait modeling attempted to better understand the relationships between λ_S_, λ_L_, and actual fall risk. With a 2-D passive model, Su and Dingwell (Su and Dingwell, 2007) showed that short-term stability λ_S_ increased linearly with the mean amplitude of applied perturbations, but not λ_L_, which remained unchanged. With an improved 3-D active model, they subsequently showed that λ_S_ was responsive to noise amplitude applied to the lateral step controller, while λ_L_ was not responsive (Roos and Dingwell, 2010). Interestingly, contrary to human results, λ_L_ was around zero, which may indicate an attractor with narrow limits: the authors explained that “the noise applied to the controller was dampened out quickly” (Roos and Dingwell, 2010). An independent study, based on 2-D passive modeling and using alternative methods to induce perturbations to the gait model, confirmed that λ_S_ relates to the probability of falling (Bruijn et al., 2011). Moreover, they observed only a weak relationship between λ_L_ and actual stability.

Two recent human studies further analyzed the use of LDS as an index for global stability and falling risk by inducing perceptual perturbations to healthy individuals. Van Schooten et al. (2011) used galvanic vestibular stimulation to impair balance. They confirmed that λ_S_ could be used to assess global stability of gait. However, they reported that the impaired balance decreased λ_L_ (improved stability). They explained that that “may be due to compensatory changes, which occur at longer timescales […].” The same contradictory stability outcome has been described in (McAndrew et al., 2011): by inducing visual and mechanical perturbations to healthy individuals, they observed increased λ_S_ and decreased λ_L_. They showed divergence curves shifting up and to the left under destabilizing conditions, with a steeper slope in the short term (higher λ_S_), and then a flatter (lower λ_L_), and higher plateau in the long term. The authors explained that the divergence curves reached their maximum local divergence limits more quickly during perturbed walking.

Taking into consideration this short review of the literature and the results of the present study, we propose that a parallel should be made between the fractal-like, persistent fluctuation pattern that is observed among consecutive strides (Terrier et al., 2005; Dingwell and Cusumano, 2010; Terrier and Dériaz, 2011) and the positive long-term LDS. In other words, as motor control allows deviations from the mean to persist across strides (α > 0.5), that translates into a long-term local instability (positive λ_L_). On the contrary, when motor control tightly regulates gait parameters, for instance by attempting to synchronize with RAC, the persistent pattern is replaced by oscillations around the target value [anti-persistence (Terrier and Dériaz, 2012)], more stationary time series take place (Terrier, 2012), and the local divergence is more quickly dampened within an attractor exhibiting narrower bounds (Figures 2, 4), as in the gait models (Roos and Dingwell, 2010). On the other hand, we hypothesize that short-term LDS λ_S_ is more related to rapid automated/unconscious motor processes that hinder uncontrolled growth of small perturbations and manage obstacle avoidance (Weerdesteyn et al., 2004). That would explain why λ_S_ is a relevant proxy for fall risk (Roos and Dingwell, 2010; Bruijn et al., 2011, 2013; McAndrew et al., 2011; Van Schooten et al., 2011). The opposite response of λ_S_ and λ_L_ (McAndrew et al., 2011; Sloot et al., 2011) is therefore more likely due to compensatory mechanisms: by altering rapid feedback mechanisms, perceptual perturbations induce not only lower short-term LDS (higher λ_S_), but also a more cautious, voluntary controlled gait, which results in higher long-term LDS (lower λ_L_), as induced by RAC.

### Correlations between local dynamic stability and statistical persistence

The global examination (*N* = 120, Figure 5) of the relationship between divergence exponents (λ) and scaling exponents (α) revealed that only long-term LDS exhibited a relevant positive correlation with statistical persistence, because both variables are highly sensitive to RAC. The assessment of the correlations under each combination of the independent variables (3 speeds and 2 conditions), would be difficult to interpret: this is why we use a multivariate approach. The results of the CCA confirmed that a relevant correlation exists between statistical persistence and long-term LDS, even when the results are separately analyzed for both conditions (*N* = 60) and corrected for speed effects. That means that individuals, who presented a more persistent pattern in stride-to-stride fluctuations tend to also have lower long-term LDS (higher λ_L_). The relationship is stronger under the treadmill + RAC condition. In this case, individuals that presented a more anti-persistent pattern in stride-to-stride fluctuation (lower α) tended to have higher long-term LDS (lower λ_L_). A previous study (Terrier and Dériaz, 2011) also showed a moderate correlation between α-ST and λ_L_ (*r* = 0.28 and 0.42), which was not significant due to a smaller sample size. An independent study also observed a strong correlation between long-term LDS and statistical persistence during treadmill walking (*r* = 0.72; Jordan et al., 2009). Overall, these results reinforce the hypothesis that both long-term instability (positive λ_L_) and statistical persistence/anti-persistence are the manifestation of a common underlying motor control process. In contrast, λ_S_ seemed not correlated with statistical persistence (Table 1), which corroborates the hypothesis that short-term LDS is related to an independent motor control process.

### Significance for neurorehabilitation

There is conclusive evidence that synchronizing gait to external clues substantially modifies the stride-to-stride fluctuation dynamics (Terrier et al., 2005; Dingwell and Cusumano, 2010; Sejdic et al., 2012; Terrier and Dériaz, 2012), the stationarity of gait parameters (Terrier, 2012) and the long-term LDS (Figures 1, 3 and Sejdic et al., 2012). It is very likely that those substantial modifications are induced by the mobilization of specific cortical sensory-motor synchronization mechanisms (Halsband et al., 1993; Zijlstra et al., 1995; Egerton et al., 2011), which partially replace (or add to) the automated regulation of the gait. This activation could be one of the underlying mechanisms that explains the benefits of cued walking in patients with neurological disorders. For instance, in PD patients, it has been shown that a combination of attentional strategy (focusing on big steps) and RAC reduced gait variability (Baker et al., 2008), which corroborates with the hypothesis that cued walking redirects higher cognitive functions to gait, and thus compensates for automated gait regulation deficit. As a result, the abovementioned parameters should be assessed in order to evaluate neurological gait disorders and the outcome of cued walking intervention. In particular, an enhanced long-term LDS could indicate a more cautious gait (compensatory mechanism), as well as the presence of a less correlated pattern (lower α) in stride-to-stride fluctuations (Herman et al., 2005).

One could wonder whether the cognitive processes that RAC mobilizes divert motor control from performing gait stabilization tasks, which may results in higher fall risk. Indeed, it is well-established that dividing attention between gait and a cognitive task may impair gait stability (dual tasks paradigm; Woollacott and Shumway-Cook, 2002; Weerdesteyn et al., 2003). It has been recently observed that combining treadmill walking and RAC requires high attentional demands (Peper et al., 2012). However, opposite to classical dual-task situations, during cued walking, the increased attentional demands are devoted to a specific gait control task. Therefore, it could be assumed that the increased control over the gait would lead to a higher level of stability and thus to lower falling risk, which could benefit patients with gait disorders. This hypothesis is confirmed by our results: both treadmill (Terrier and Dériaz, 2011) and RAC tend to enhance LDS, or at least do not diminish it (Sejdic et al., 2012). Similarly, a recent study analyzed the influence of RAC on obstacle avoidance capabilities in PD patients (Nanhoe-Mahabier et al., 2012). The experimental design combined treadmill walking and RAC, as in the present study. The authors observed that PD patients were able to successfully execute an obstacle avoidance task, when auditory cueing is administered simultaneously. They concluded: “our data suggest that PD patients can benefit from auditory cueing even under complex, attention-demanding circumstances, and that the metronome does not act as a dual task that negatively affects gait.”

## Conclusion

Synchronizing steps with rhythmic auditory stimuli tends to induce more dampening in the divergences among state space trajectories, which likely reflects a more restricted range in the gait dynamics. That effect is concomitant to the apparition of a strong anti-persistent pattern in the stride-to-stride fluctuations of gait parameters. Both phenomena are probably the manifestation of a more conscious/voluntary modality of gait control.

Furthermore, although further studies are needed to analyze LDS and other variability indexes in various neurological gait disorders, the present study introduced new evidence that cued walking could be a valuable and safe treatment in gait rehabilitation.

### Conflict of interest statement

The authors declare that the research was conducted in the absence of any commercial or financial relationships that could be construed as a potential conflict of interest.

## Figures and Tables

**Figure A1:**
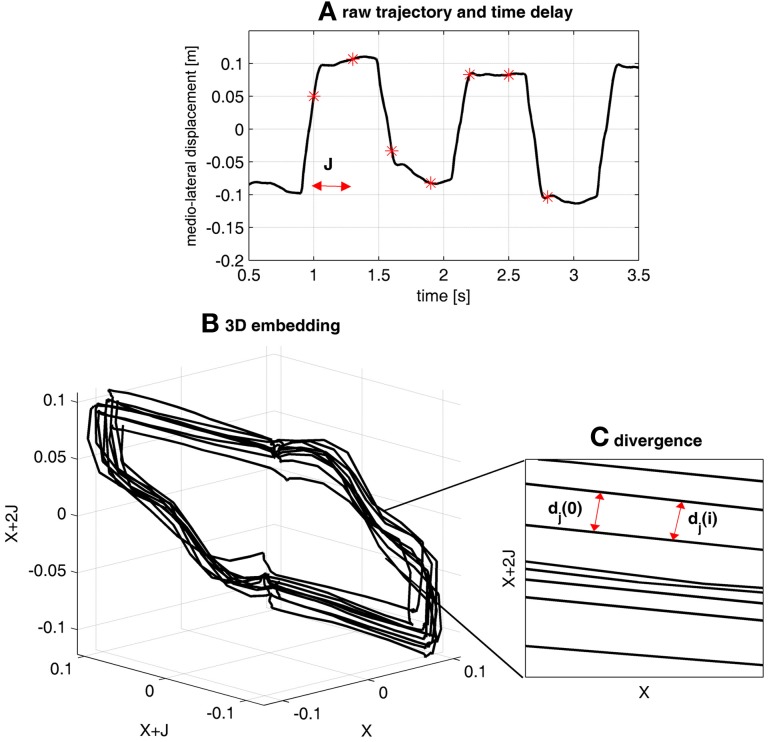
**Illustration of the computing of LDS with gait data**. **(A)** Shows 3.5 s of the medio-lateral displacement of the center of pressure sampled at 50 Hz. The result of the average mutual information analysis was 18 samples (0.36 s), which defined the reconstruction delay J. The results of the false neighbor analysis revealed that the minimal embedding dimension should be 6. Therefore, five time-delayed copies of the original time series have been built, according to the Equation 2. **(B)** Displays the reconstructed attractor with *J* = 18 and *m* = 6. Only the first three dimensions are used to build a 3D projection of the 6D attractor. **(C)** Is a 2D magnification of the attractor. It shows the flow of the trajectories along which the maximal Lyapunov exponent will be computed. Two nearest neighbors (Equation A3) are shown, separated by the initial distance d_*j*_(0). Two downstream points are separated by the distance d_*j*_(i) (Equation A4).

## Supplementary material

Movie 1 **Illustration of the measurement of the center of pressure**. Five seconds of analysis are shown, slowed down to 25 s. The pressure on the grid of sensors is shown with color ranging from blue (no pressure) to red (high pressure): the succession of 10 steps is visible. The dark red circle indicates the current position of the center of pressure. The dark red trace shows the corresponding trajectory, which is also displayed in Figure 1.

## Appendix A: maximal lyapunov exponent

The objective of this appendix is to summarize the theoretical background behind the assessment of the local dynamic stability (LDS) based on the Rosenstein's algorithm. Most of the concepts described below are adapted from Rosenstein et al. (1993), Fraser and Swinney (1986), and Kennel et al. (1992).

### Lyapunov exponents

The Lyapunov exponents assess the sensitivity to initial conditions of a dynamical system, which is characterized by a finite number of *n* state variables and *n* equations. If an *n*-dimensional sphere of initial conditions is defined, the exponential rates of divergence are given by a spectrum of *n* Lyapunov exponents (λ). These exponents describe a multidimensional ellipsoid expending/contracting with time along particular axes (Lyapunov directions), which are dependent upon the system flow. A particular Lyapunov exponent represents the local instability in a given direction. If the exponent is positive, it diagnoses chaos. When the system is globally stable the rate of contractions in some directions must counterbalance the rate of expansions in others in order to obtain a stable attractor. Thus, the sum across the entire spectrum of the Lyapunov exponents is negative. The largest (maximal) Lyapunov exponent (λ_1_ ≥ λ_2_ ≥ … ≥ λ_*n*_) defines the direction along which the system is the most unstable, because the exponential growth in this direction will dominate growth along the other directions. It is defined by the following equation:

(A1) $$d(t)=Ce^{\lambda_{1}t}$$

*d(t)* being the average divergence at time *t* and C a constant that normalize the initial separation.

### Phase space reconstruction: embedding

When working with real world data, the set of *n* equations characterizing a dynamical system is not available. However, the attractor dynamics can be reconstructed from a single time series according to the Takens's theorem (Takens, 1980). The process that unfolds the time series into a multidimensional state space is referred to as embedding. The reconstruction is based on the time delay method. The state of the system *X* defined by a *N*-point time series {*x*_1,_*x*_2_…, *x*_*n*_} at discrete time *i* is:

(A2) $$X_{i}=(x,x_{i+J},\ldots ,x_{i+(m-1)J})$$

Where *J* is the lag or the reconstruction (or time) delay and *m* is the embedding dimension. In principle, for an infinite noise-free time series, the time delay *J* and the embedding dimension *m* can be arbitrarily chosen. In smaller data set of noisy data, a too small *J* would produce a compressed attractor along the identity line (high correlation or excessive redundancy); conversely, a too large *J* would produce causally disconnected attractor (irrelevance issue) (Fraser and Swinney, 1986; Kim et al., 1999). A solution is to choose a delay *J* that maximizes the independence between the coordinates (for instance *x*_*i*_ and *x*_*i* + *J*_). A linear independence is realized when the autocorrelation function of the time series first pass through zero. However, non-linear dependence is likely in most dynamical systems. The method of the AMI (Fraser and Swinney, 1986) solves this issue by assessing the general dependence of two variables (namely, *x*_*i*_ and *x*_*i* + *J*_) using the Shannon's information theory. *J* is determined from the first minimum in the mutual information function of the time series. Thus, *x*_*i* + *J*_ adds the largest amount of information available to the first coordinate *x*_*i*_ and conserves some correlation among them, which has been empirically proven to be a good equilibrium between redundancy and irrelevance (Fraser and Swinney, 1986).

The issue of the choice of a correct embedding dimension is more topological than dynamical. A phase space with a sufficient number of dimensions allows the attractor to deploy without self-crossing, thus correctly capturing the dynamic flow of the system. However, an excessive number of dimensions make difficult the manipulation of the reconstructed attractor (high computational time). Furthermore no dynamics is operating in the dimensions in excess and they are mostly populated by noise. Conversely, a too small number of dimensions increases the risk of spurious vicinity between points in the phase space due to projection effects. In other words, the distances between points are distorted. Thus, a too compact attractor contains a high number of these false neighbors, which makes difficult to account for the true flow of the system. The method of GFNN assesses the rate of spurious vicinity that occurs in under-dimensioned phase spaces (Kennel et al., 1992). It differentiates the points that are neighbor due to the dynamics of the system (following the flow) from the points that are neighbor solely due to projection issues. The percentage of false neighbors is computed for increasing dimensions, and the minimal dimension for a correct embedding is found when the percentage is close to zero.

### Maximal Lyapunov exponent

The rate of exponential divergence in the appropriately embedded attractor is computed as follows: First, nearest neighbor of each point on the attractor is located. The nearest neighbor $X_{\hat{j}}$ is found by searching the point that minimizes the distance to the particular reference point*X*_*j*_:

(A3) $$d_{j}(0)=\min\text{​}\|X_{j}-X_{\hat{j}}\|$$

where *d*_*j*_(0) is the initial distance from the *j*th point to its nearest neighbor, and ∥ ∥ denotes the Euclidian norm. An additional constraint is imposed, namely that the nearest neighbors must be separated by a time, which exceeds the mean period of the time series. As a result, the two neighbors are on a different orbit of the attractor. Then the Euclidian distances *d*_*j*_(*i*) between the two trajectories defined by the subsequent points *i* downstream the reference point and its nearest neighbor are computed, for instance as follows for the first iteration:

(A4) $$d_{j}(1)=\|X_{\left(j+1\right)}-X_{\left(\hat{j}+1\right)}\|$$

It is expected that the rate of divergence between the trajectories is given by the Equation A1. In its logarithm form, the Equation A1 becomes:

(A5) $$\ln d_{j}(i)\approx \ln C_{j}+\lambda_{1}(i\Delta t)$$

where *C*_*j*_ is the initial separation. This linear equation defines a set of parallel lines with slope equal to the maximal Lyapunov exponent. Thus, the average logarithmic divergence for all *j* is computed <ln *d*_*j*_(*i*) ≥ *avg*[*d*_1_(*i*), *d*_2_(*i*), …, *d*_*n*_(*i*)]. The average logarithmic divergence <ln *d*_*j*_(*i*)> can be represented in a diagram as a function of time (see Figures 2, A2). Finally, the Lyapunov exponent is approximated using least-square fit to the average line:

(A6) $$y(i)=\frac{1}{\Delta t}\langle \ln d_{j}(i)\rangle$$

### Divergence curves and application to gait dynamics

Using the example of the Lorenz attractor, Rosenstein et al. described that plotting the average divergence as a function of time (Figure A2) showed “a long linear region” after a “short transition.” A plateau occurs at longer times (“saturation”) because the attractor is bounded in phase space, which implies that the average divergence cannot continuously grow with time. Thus, the linear fit to the divergence must be performed on “prominent” linear region. The authors acknowledge that the determination of that region is mostly empirical and may be difficult in real systems.

Gait dynamics produces a divergence curve that does not exhibit a clear linear region (Figure A2). Whether gait could be considered as a real chaotic process described by a limited set of equations is therefore debatable. However, a pragmatic approach is to consider that the linear fitting estimates the local divergence over a given time scale, which is used to characterize the dynamics of the system, even if a true maximal Lyapunov exponent does not exist (Dingwell, 2006). Thus, the term “divergence exponent” may be more appropriate than the term “maximal Lyapunov exponent.” Each time the foot is on the ground, it gives to the motor control the opportunity to thwart perturbations; consequently, the rate of divergence per stride (or per step) is more physiological than the rate of divergence per second (Bruijn et al., 2013). The long-term divergence fitted against a time scale between the fourth and the tenth stride has been adopted on a purely empirical basis (Dingwell and Cusumano, 2000). On the other hand, the short-term divergence fitted over the first stride (or first step) estimates the local dynamic stability immediately after a perturbation, which is more physiologically sound.

## Appendix B: detrended fluctuation analysis (DFA)

The aim of this appendix is to briefly summarize the DFA method and to report the scaling exponents, which were showed in the companion article (Terrier and Dériaz, 2012).

### Background

A process that possesses a “memory” of its previous comportment (feedback loop) exhibits autocorrelations in its output. The correlation function *C(s)* indicate the degree of correlation among subsequent data points in the time series separated by a time lag (or scales) *s* (Kantelhardt et al., 2001). *C(s)* is by definition 0 for all *s* if the time series is uncorrelated (white noise). If the time series presents correlations over short time scales, *C(s)* declines exponentially fast as *C*(*t*) ~ exp(−*s*/*t*_*x*_)(*t*_*x*_ being the decay time), which is typical for a simple autoregressive process (AR). In case of long-range correlations, the slow decline follows a power law:

(B1) $$C(s)\propto s^{-\gamma }$$

with the correlation exponent 0 < γ <1. Such persistent behavior is typical for a self-affine, fractal-like scaling behavior, i.e., autoregressive fractionally integrated moving average model (ARFIMA). The estimation of *C(s)* is problematic if many non-stationarities are present in the time series. Therefore, many alternative methods have been proposed to estimate the correlation exponent, such as the Hurst's Rescaled-Range analysis, or the spectral analysis.

### DFA

The detrended fluctuation analysis has been designed to detect the presence of long-range correlations in time series, which are highly unstationary. The time series of length *N* is first integrated. Then, it is divided in non-overlapping boxes of equal length *n*. In each box, a linear fit (least squares method) is performed. The average fluctuation *F(n)* for that box length is determined as follows:

(B2) $$F(n)=\sqrt{\frac{1}{N}\sum_{k=1}^{N}{\left[y(k)-y_{n}(k)\right]}^{2}}$$

where *y*_*n*_(*k*) is the y-coordinate of the kth point of the straight line resulting of the linear fit, and *y(k)* is the corresponding point in the original time series. The procedure is repeated for increasing box size *n*, over all time scales. It is expected that the fluctuation *F(n)* increases with box size *n*. If the time series exhibits long-range power-law autocorrelations, the relationship between *F(n)* and *n* would be *F*(*n*) ≈ *n*^α^. Thus, the scaling exponent *α* is determined by a linear fit of *F(n)* vs. *n* in a log-log graph. If α = 0.5, then a random process (uncorrelated white noise) is most likely. If 0.5< α < 1, a persistent pattern is present in the times series, most likely characterized by long-range correlations. If 0< α < 0.5, an anti-persistent pattern is likely.

### DFA results

The Table B1 summarizes the results of our previous study, which are used in the correlation analyses.

**Table B1:** **DFA results**.

***N* = 20**		**Treadmill**		**Treadmill + RAC**		**Relative change (%)**
Slow	α-ST	0.85	(0.11)	*0.27*	*(0.11)*	−68
	α-SL	0.79	(0.11)	*0.33*	*(0.10)*	−58
	α-SS	*0.23*	*(0.08)*	*0.29*	*(0.08)*	26
PWS	α-ST	0.80	(0.12)	*0.28*	*(0.11)*	−65
	α-SL	0.72	(0.11)	*0.36*	*(0.08)*	−50
	α-SS	*0.31*	*(0.08)*	*0.30*	*(0.06)*	−3
Fast	α-ST	0.75	(0.11)	*0.32*	*(0.12)*	−57
	α-SL	0.72	(0.11)	*0.39*	*(0.11)*	−46
	α-SS	*0.30*	*(0.08)*	*0.30*	*(0.06)*	0

The detrented fluctuation analysis (DFA) was used to compute scaling exponents (α), which evaluate the statistical persistence present in the time series of stride time (ST), stride length (SL) and stride speed (SS). Both conditions were tested: treadmill only and treadmill with rhythmic auditory cueing (RAC). Values are means and (SD). Values for anti-persistent dynamics are shown in italic. The last column shows the relative change between both conditions. PWS, preferred walking speed.
